# Global Trends in the Research of Ulcerative Colitis Treatments (2021–2025): A Bibliometric Review

**DOI:** 10.7759/cureus.91262

**Published:** 2025-08-29

**Authors:** Jorge Zapatier, Ethan Nichols, Vindhya N Reddy, Latha Ganti

**Affiliations:** 1 Department of Biology, Orlando Science High School, Orlando, USA; 2 Department of Biochemistry, Colgate University, Windermere, USA; 3 Department of Medicine, Apollo Institute of Medical Sciences and Research, Hyderabad, IND; 4 Medical Science, The Warren Alpert Medical School of Brown University, Providence, USA; 5 Research, Orlando College of Osteopathic Medicine, Winter Garden, USA; 6 Emergency Medicine and Neurology, University of Central Florida, Orlando, USA

**Keywords:** bibliometric analysis, gastroenterology, global research trends, inflammatory bowel disease, treatments, ulcerative colitis (uc)

## Abstract

Ulcerative colitis (UC) is a chronic idiopathic inflammatory bowel disease characterized by mucosal ulceration and inflammation confined to the colonic epithelium. While colonoscopy remains the diagnostic gold standard, fecal calprotectin (FC) is a validated non-invasive biomarker. Therapeutic management encompasses immunosuppressive agents including anti-TNF biologics such as infliximab, vedolizumab, and ustekinumab for severe disease manifestations, whereas mild-to-moderate UC is managed with variable dosing regimens of 5-aminosalicylates administered via oral or rectal routes. This bibliometric analysis utilized the Web of Science (WoS) database to extract publication data spanning from January 1, 2021, to June 14, 2025, with subsequent exportation of tab-delimited datasets to VOSviewer (version 1.6.20) for comprehensive bibliometric visualization and analysis. Bibliometric analysis demonstrated that the People's Republic of China exhibited the highest publication output with predominantly recent scholarly contributions. Therapeutic research predominantly focused on biologic agents, specifically infliximab (677 publications), vedolizumab (269 publications), and ustekinumab (234 publications) over the five-year study period. Temporal analysis utilizing the WoS Core Collection revealed consistent publication growth, with 2024 demonstrating peak research output. Categorical distribution analysis indicated "Gastroenterology Hepatology" as the predominant research domain.

## Introduction and background

Ulcerative colitis (UC) is a chronic idiopathic inflammatory bowel disease (IBD) characterized by mucosal ulceration and inflammation [1,2]. UC is the most common form of IBD, manifesting clinically through bloody stool, increased bowel movement, abdominal cramping, and fecal incontinence [3]. While colonoscopy remains the diagnostic gold standard for UC, non-invasive biomarkers such as fecal calprotectin (FC) provide valuable diagnostic alternatives [4,5].

The pathogenesis of UC remains incompletely elucidated; however, contemporary evidence supports an autoimmune etiology involving dysregulated immune responses to commensal gut microbiota. This aberrant immunological response is mediated through dendritic cells and intestinal epithelial cells, resulting in the excessive production of pro-inflammatory cytokines, including interleukin-5 (IL-5), interleukin-13 (IL-13), and tumor necrosis factor-alpha (TNF-α), ultimately leading to mucosal erosion and ulcerative lesions [6]. Multiple environmental factors contribute to UC development, encompassing dietary patterns, antecedent infectious processes, and antibiotic exposure [7].

Epidemiological data demonstrate a positive correlation between industrialization and UC incidence rates since 1990, with highly industrialized nations experiencing disproportionately elevated disease burden [5]. The United States, among the world's most industrialized countries, reports approximately 1.5 million individuals affected by UC, underscoring the significant public health impact of this condition.

Contemporary UC management follows a severity-stratified therapeutic approach as outlined in clinical guidelines [4]. For mild UC, oral 5-aminosalicylates (5-ASA, ≥2g/day) serve as first-line therapy, with rectal 5-ASA (1g/day) and budesonide MMX utilized when initial therapy fails. This approach is particularly preferred for proctitis and left-sided UC, with combined rectal and oral therapy demonstrating optimal efficacy [4,5]. Moderate UC management involves higher-dose oral 5-ASA (up to 4.8 g/day), budesonide MMX, and systemic corticosteroids when necessary, with careful monitoring for steroid dependence as treatment intensity escalates. Severe UC requires aggressive intervention including intravenous corticosteroids; biologic agents such as infliximab, adalimumab, and golimumab; and JAK inhibitors like tofacitinib [4,5]. Hospitalization is frequently necessary for severe cases, with surgical evaluation for patients who fail to respond to intensive medical therapy [3]. The table outlines the stepwise treatment approach for UC, with therapy intensifying based on disease severity. Notably, severe cases often require hospitalization and advanced therapies such as biologics or JAK inhibitors (Table 1).

**Table 1:** Treatment options by disease severity Treatment options for ulcerative colitis (UC) stratified by disease severity. Recommendations include escalating therapies from oral 5-aminosalicylates in mild UC to intravenous corticosteroids, biologics, and JAK inhibitors in severe cases. Clinical notes highlight preferred strategies and when to consider escalation or hospitalization.

Severity	Treatment options	Notes
Mild UC	Oral 5-aminosalicylates (5-ASA, ≥2 g/day) - 5-ASA (1 g/day) - Budesonide MMX (if 5-ASA fails)	Preferred for proctitis and left-sided UC; rectal+oral combo is the best.
Moderate UC	Higher-dose oral 5-ASA (up to 4.8 g/day) - Budesonide MMX - Systemic corticosteroids (if needed)	Step-up if mild therapies fail; watch for steroid dependence.
Severe UC	IV corticosteroids - Biologics (infliximab, adalimumab, golimumab) - JAK inhibitors (tofacitinib)	Hospitalization often needed; assess for colectomy if no response.

Given the complexity of UC management, the authors conducted a comprehensive bibliometric analysis to collate current treatment modalities and identify emerging therapeutic approaches for UC.

## Review

Methods

Search Strategy

This bibliometric analysis was conducted using the Web of Science (WoS) Core Collection, a multidisciplinary citation database that provides comprehensive coverage of biomedical and clinical literature dating back to 1990. WoS was chosen as the primary data source because of its well-structured citation indexing, consistent metadata formats, and established utility in bibliometric research, which facilitates reliable analysis of citation networks and research trends. Other databases such as PubMed and Scopus were not included in this analysis, as their citation architecture and compatibility with bibliometric software such as VOSviewer are comparatively limited for large-scale citation network visualization and co-occurrence mapping [8]. The search was carried out on June 14, 2021, at 10 am Eastern Standard Time (EST). 

The search was carried out using the following Boolean query: “Ulcerative Colitis” AND “Treatments”. To enhance specificity and reduce the retrieval of irrelevant articles, search filters were applied. The time frame was restricted to publications from January 1, 2021, to June 14, 2025, thereby capturing the most recent five years of scholarship. This strategy yielded a total of 9,492 unique publications. The search was performed without restrictions on document type, thereby including original research articles, reviews, proceedings papers, and other scholarly outputs indexed in WoS. The search was limited to the English language.

Data Extraction

Standard bibliometric methods were used for data extraction [9]. Following retrieval, bibliographic records were exported from WoS using the tab-delimited text format to ensure compatibility with our chosen bibliometric analysis software, VOSviewer. Exported fields included titles, author names, institutional affiliations, countries of origin, publication years, source journals, abstracts, keywords, and citation counts. These metadata elements formed the basis for subsequent analyses of authorship patterns, collaboration networks, keyword trends, and geographical contributions.

Data integrity was confirmed through a multi-step cleaning process. Duplicate records, arising from indexing overlaps across WoS sub-collections, were systematically removed. Author names were normalized to reduce variability in citation spellings (e.g., “J Lopez” vs. “Lopez, Jorge”). Similarly, institutional affiliations were standardized to account for naming inconsistencies (e.g., “Univ of Florida” vs. “University of Florida”). This normalization process ensured accurate representation of authors, organizations, and collaborative networks.

The cleaned dataset was then imported into VOSviewer version 1.6.20, a widely used bibliometric visualization software designed for network-based analysis. VOSviewer’s ability to construct co-authorship, co-citation, and keyword co-occurrence networks made it particularly suitable for the present study, which aimed to characterize global trends in UC treatment research.

Analysis

The analysis was structured around three primary dimensions: organizational contributions, geographical distribution, and thematic keyword trends.

Organizational Analysis

Co-authorship networks were generated to evaluate the patterns of institutional output. To reduce visual clutter and emphasize the most influential contributors, only organizations with a minimum of 70 documents were included in the visualization. This threshold ensured that analyses focused on institutions with sustained productivity in UC treatment research, allowing for the identification of global centers of excellence and collaboration hubs.

Country-Level Analysis

A separate co-authorship network was constructed to evaluate international research contributions. Here, a stricter threshold of 50 publications per country was applied. This allowed the visualization to highlight major national contributors, collaborative partnerships between countries, and geographical trends in research production. Special attention was given to cross-border collaborations, as these often signal large-scale clinical trials, multinational guideline development, and transdisciplinary innovation.

Keyword Co-occurrence Analysis

To characterize evolving research themes, a co-occurrence analysis of author keywords was performed. A minimum threshold of 100 keyword occurrences was established, ensuring that only terms with substantial frequency were visualized. This method allowed for the identification of dominant research themes (e.g., biologics, immunotherapy, microbiome modulation) and emerging areas of focus in UC treatments. Temporal overlay visualization further enabled assessment of the chronological evolution of these themes, distinguishing between long-established topics and novel therapeutic directions.

Visualizations were generated using VOSviewer’s clustering algorithms, which group related nodes (authors, institutions, countries, or keywords) based on similarity measures such as co-occurrence frequency. Network maps were color-coded by cluster to enhance interpretability, and node sizes were scaled according to frequency or citation impact. These bibliometric maps provided an intuitive representation of the structure, development, and interdisciplinary connections within UC treatment research.

To ensure reproducibility and methodological rigor, analytical decisions-including threshold settings, database selection, and visualization techniques-were guided by prior bibliometric research standards and recommendations. This approach aligns with the Equator network's preliminary guideline for reporting bibliometric reviews of the biomedical literature [10].

Results

**Figure 1:**
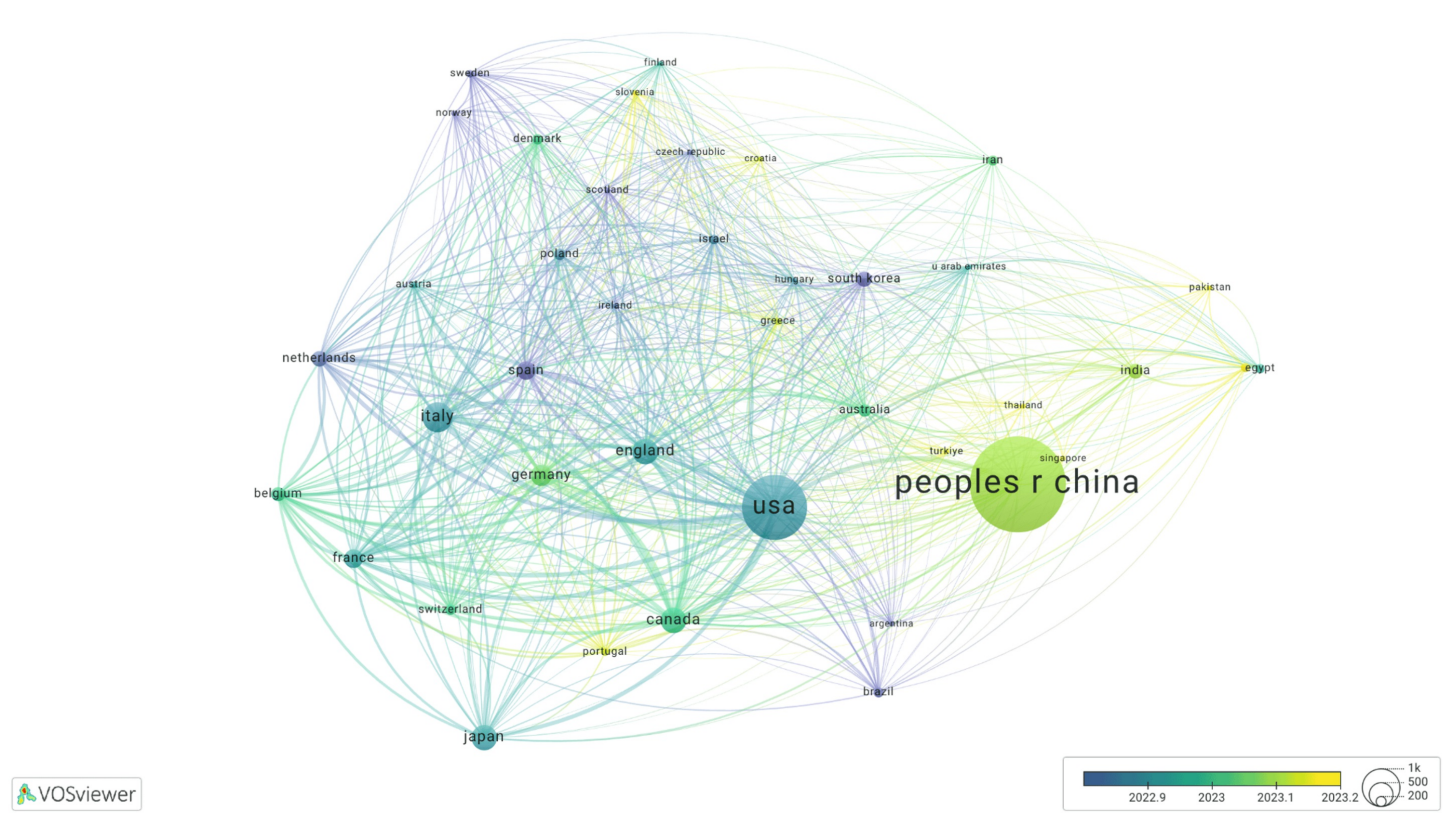
Visualization of publications by countries This VOSviewer visualization displays collaborative relationships between countries based on co-authorship in ulcerative colitis (UC)-related publications. Node size reflects the number of publications, line thickness indicates collaboration strength, and color gradient corresponds to the average year of publication. China and the USA appear as the most prolific contributors.

The People's Republic of China has produced the most articles and the most recent ones (Figure 1). Countries considered developing by the IMF exhibit a trend of more recent publications, such as those from Pakistan, Egypt, and India. The top four countries with the highest publication output are the People's Republic of China, the United States, Italy, and England, with 3,115, 1,936, 738, and 604 publications, respectively.

**Figure 2:**
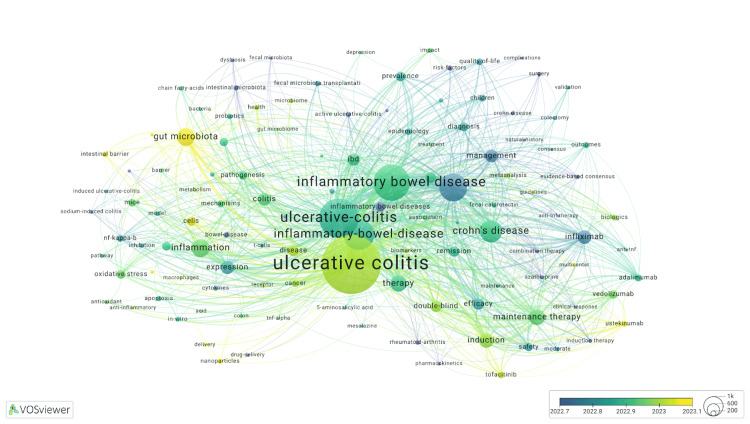
Visualization of the most frequent keywords in the publications VOSviewer keyword co-occurrence network for publications on ulcerative colitis. The node size represents the frequency of keyword occurrence, while the color gradient reflects the average publication year. Lines indicate co-occurrence links between keywords.

The keyword analysis revealed "ulcerative colitis" appearing with "ulcerative-colitis" 645 times, "inflammatory bowel disease" appearing with "inflammatory-bowel-disease" 418 times, and "Crohns-disease" appearing with "Crohn's disease" 484 times (Figure 2). Excluding these keywords due to redundancy, the top four keywords were "inflammation", "gut microbiota", "maintenance therapy", and "infliximab", with 943, 931, 810, and 677 publications, respectively. Among the 9,492 publications, the terms "ulcerative colitis" (N = 4,214) and "ulcerative-colitis" (N = 2,553) were the most prevalent, followed by "inflammatory bowel disease" (N = 2,289) and "inflammatory-bowel-disease" (N = 1,853), with "Crohns-disease" (N = 1,639) and "Crohn's disease" (N = 1,234) also showing significant prevalence.

**Figure 3:**
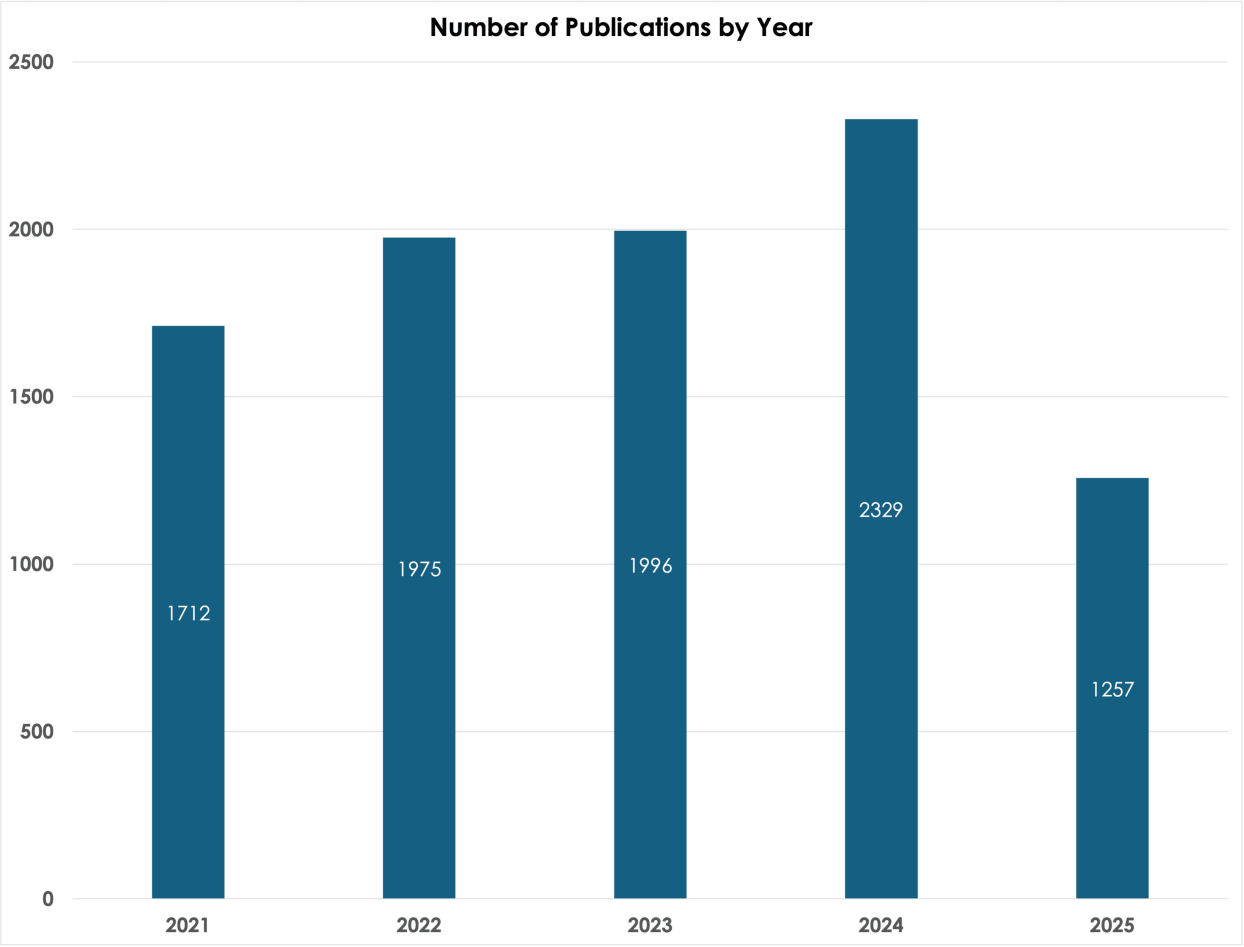
Bar graph depicting the number of publications per year in ulcerative colitis

The bar graph illustrates the annual volume of publications globally, demonstrating that 2024 had the highest number of articles published concerning UC treatments (Figure 3). There were 1,712 publications in 2021, 1,975 publications in 2022, 1,996 in 2023, and 2,329 in 2024. In 2025, there were only 1,257 publications. Considering that all publications regarding the treatment of UC have not been completely updated and or finished, the trend of a consistent rise in publications over the past five years cannot be supported although it could be inferred. 

**Figure 4:**
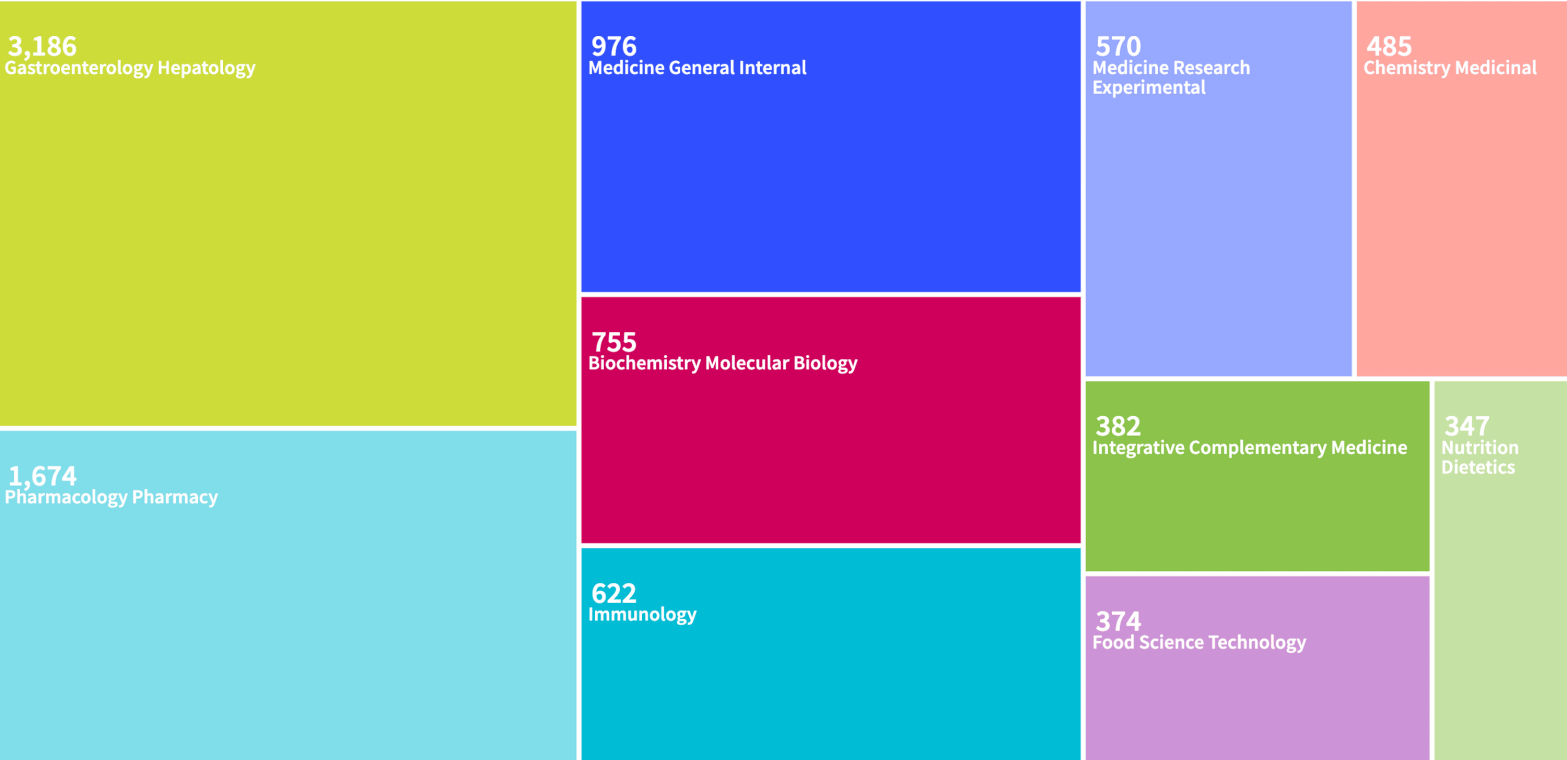
Tree map showing the categorization of publications in Web of Science Tree map of ulcerative colitis (UC) publications by Web of Science categories. The size of each block represents the relative number of publications within that subject area, with larger blocks indicating higher publication volumes.

This tree map is created using the WoS to analyze the distribution of publications by category. The WoS generalizes these categories to specific topics (Figure 4). According to the graph, the most significant number of publications is categorized in Gastroenterology Hepatology, with 3,116 publications. The second highest in number of publications (1,643) was published in Pharmacology Pharmacy, and the third most was from Medicine General Internal, with 950 publications. 

Discussion

Figure 1 illustrates that China has the highest number of publications and the most recent research output among all countries analyzed. This observation suggests that Chinese researchers have been increasingly inclined to publish on UC therapeutics, likely due to the rising prevalence of UC in China. The increasing incidence of UC in China has been attributed to dietary and cultural transitions toward Western lifestyle patterns [11,12]. Similarly, the United States demonstrates substantial research output, which may be attributed to elevated UC prevalence associated with dietary factors, genetic predisposition, and cultural influences [13-15].

Figure 2 reveals frequently occurring keywords including inflammation, gut microbiota, maintenance therapy, and infliximab. The most extensively studied therapeutic agents are infliximab (677 publications), vedolizumab (269 publications), and ustekinumab (234 publications). The continued research focus on infliximab reflects its established status as the gold standard for treating UC, attributed to its efficacy as an immunosuppressive agent and anti-inflammatory properties [16,17]. The substantial research output on vedolizumab indicates growing academic interest in this therapeutic option, supported by its demonstrated safety and efficacy profile in the management of UC [18]. The emergence of ustekinumab-focused studies reflects recent research interest following its FDA approval for UC in October 2019, particularly as an alternative therapeutic option for patients who demonstrate an inadequate response to anti-TNF agents, such as infliximab [19].

Excluding 2025 data due to an incomplete dataset, Figure 3 illustrates a positive trend in publication volume across the study period. This growth pattern reflects sustained research momentum following the documented 18% decline in non-COVID-19-related publications in 2020 [20]. This trend highlights the growing interest in UC research, driven by the increasing prevalence of UC in developing nations and the knowledge gaps surrounding its pathophysiology, which continue to challenge the research community [21,22].

Limitations

This study is limited by the fact that only articles written in English are included in the analysis and data. This reduces the total number of studies available to the authors about UC, as papers published in non-English-speaking nations are not included in the search. Another limitation is the exclusive reliance on data obtained from the Web of Science, meaning any studies on UC that exist solely in other databases, such as Scopus or another major database, were not included. In addition, the search was only conducted for the years 2021-2025, which is a relatively short date range, significantly narrowing the results in this search.

## Conclusions

This bibliometric analysis shows a significant growth in research on UC treatment globally, with China leading in publication volume (3,115 articles), followed by the United States (1,936). Research emphasis remains concentrated on established treatments such as infliximab (677 publications), concurrent with intensified investigation of novel biologics including vedolizumab and ustekinumab. The positive growth in publications, from 1,712 in 2021 to 2,329 in 2024, reflects positive research trends driven by an escalating global disease burden. These findings underscore the international effort to advance UC therapeutic paradigms and portend continued research proliferation addressing this multifaceted inflammatory disorder. 

## References

[1] Ordás I, Eckmann L, Talamini M, Baumgart DC, Sandborn WJ (2012). Ulcerative colitis. Lancet.

[2] Eder P, Łodyga M, Gawron-Kiszka M (2023). Guidelines for the management of ulcerative colitis. Recommendations of the Polish Society of Gastroenterology and the Polish National Consultant in Gastroenterology. Prz Gastroenterol.

[3] Ungaro R, Mehandru S, Allen PB, Peyrin-Biroulet L, Colombel JF (2017). Ulcerative colitis. Lancet.

[4] Rubin DT, Ananthakrishnan AN, Siegel CA, Sauer BG, Long MD (2019). ACG clinical guideline: ulcerative colitis in adults. Am J Gastroenterol.

[5] Jostins L, Ripke S, Weersma RK (2012). Host-microbe interactions have shaped the genetic architecture of inflammatory bowel disease. Nature.

[6] de Souza HS, Fiocchi C (2016). Immunopathogenesis of IBD: current state of the art. Nat Rev Gastroenterol Hepatol.

[7] Ng SC, Shi HY, Hamidi N (2020). Worldwide incidence and prevalence of inflammatory bowel disease in the 21st century: a systematic review of population-based studies. Lancet.

[8] Falagas ME, Pitsouni EI, Malietzis GA, Pappas G (2008). Comparison of PubMed, Scopus, Web of Science, and Google Scholar: strengths and weaknesses. FASEB J.

[9] Ganti L, Persaud NA, Stead TS (2025). Bibliometric analysis methods for the medical literature. Acad Med Surg.

[10] Montazeri A, Mohammadi S, M Hesari P, Ghaemi M, Riazi H, Sheikhi-Mobarakeh Z (2023). Preliminary guideline for reporting bibliometric reviews of the biomedical literature (BIBLIO): a minimum requirements. Syst Rev.

[11] Xu L, He B, Sun Y (2023). Incidence of inflammatory bowel disease in urban China: a nationwide population-based study. Clin Gastroenterol Hepatol.

[12] Yang H, Qian J (2024). Epidemiological research, burden, and clinical advances of inflammatory bowel disease in China. Chin Med J (Engl).

[13] Kaplan GG, Bernstein CN, Coward S (2019). The impact of inflammatory bowel disease in Canada 2018: epidemiology. J Can Assoc Gastroenterol.

[14] Yamazaki M, Chung H, Xu Y, Qiu H (2023). Trends in the prevalence and incidence of ulcerative colitis in Japan and the US. Int J Colorectal Dis.

[15] Ananthakrishnan AN (2015). Epidemiology and risk factors for IBD. Nat Rev Gastroenterol Hepatol.

[16] Gisbert JP, González-Lama Y, Maté J (2007). Systematic review: Infliximab therapy in ulcerative colitis. Aliment Pharmacol Ther.

[17] Rutgeerts P, Sandborn WJ, Feagan BG (2005). Infliximab for induction and maintenance therapy for ulcerative colitis. N Engl J Med.

[18] Takatsu N, Hisabe T, Higashi D, Ueki T, Matsui T (2020). Vedolizumab in the treatment of ulcerative colitis: an evidence-based review of safety, efficacy, and place of therapy. Core Evid.

[19] Gisbert JP, Parody-Rúa E, Chaparro M (2024). Efficacy, effectiveness, and safety of ustekinumab for the treatment of ulcerative colitis: a systematic review. Inflamm Bowel Dis.

[20] Raynaud M, Goutaudier V, Louis K (2021). Impact of the COVID-19 pandemic on publication dynamics and non-COVID-19 research production. BMC Med Res Methodol.

[21] Wang J, Mao T, Zhou H, Jiang X, Zhao Z, Zhang X (2024). Global trends and hotspots of ulcerative colitis based on bibliometric and visual analysis from 1993 to 2022. Medicine (Baltimore).

[22] Porter RJ, Kalla R, Ho GT (2020). Ulcerative colitis: recent advances in the understanding of disease pathogenesis. F1000Res.

